# Ovarian cancer tumor immune profiles associated with intrauterine device and oral contraceptive use

**DOI:** 10.1038/s41416-026-03508-9

**Published:** 2026-06-27

**Authors:** Jennifer M. Mongiovi, Ana Babic, Naoko Sasamoto, Mary K. Townsend, Allison F. Vitonis, Mollie Barnard, Jonathan Hecht, T. Rinda Soong, Jose R. Conejo-Garcia, Lauren C. Peres, Joellen M. Schildkraut, Holly R. Harris, Jennifer A. Doherty, Francesmary Modugno, Brooke L. Fridley, Shelley S. Tworoger, Kathryn L. Terry

**Affiliations:** 1 Department of Epidemiology, Harvard T.H. Chan School of Public Health, Boston, MA USA; 2 Department of Obstetrics and Gynecology, Brigham and Women’s Hospital, Boston, MA USA; 3 Department of Medical Oncology, Dana Farber Cancer Institute, Boston, MA USA; 4 Public Health Sciences Division, Fred Hutchinson Cancer Center, Seattle, WA USA; 5 Division of Oncological Sciences and the Knight Cancer Institute, Oregon Health & Science University, Portland, OR USA; 6 Slone Epidemiology Center, Boston University Chobanian & Avedisian School of Medicine, Boston, MA USA; 7 Department of Pathology, Beth Israel Deaconess Medical Center, Boston, MA USA; 8 Department of Pathology, University of Pittsburgh School of Medicine, Pittsburgh, PA USA; 9 Department of Integrative Immunobiology, Duke School of Medicine, Durham, NC USA; 10 Department of Cancer Epidemiology, Moffitt Cancer Center, Tampa, FL USA; 11 Department of Epidemiology, Emory University, Rollins School of Public Health, Atlanta, GA USA; 12 Department of Epidemiology, School of Public Health, University of Washington, Seattle, WA USA; 13 Department of Population Health Sciences, Huntsman Cancer Institute, University of Utah, Salt Lake City, UT USA; 14 Department of Obstetrics, Gynecology and Reproductive Sciences, University of Pittsburgh, Pittsburgh, PA USA; 15 Biostatistics and Epidemiology Core, Children’s Mercy Kansas City, Kansas City, MO USA

**Keywords:** Ovarian cancer, Biomarkers, Risk factors

## Abstract

**Background:**

Non-hormonal intrauterine devices (IUDs) create an inflammatory uterine environment while oral contraceptives (OC) suppress ovulation and have different associations with ovarian cancer risk. We have evaluated the associations of these two contraceptive exposures with ovarian tumor immune infiltration.

**Methods:**

This study assessed associations of IUD and OC use with tumor immune features via multiplex immunofluorescence in 24 ovarian tumor tissue microarrays from four case-control and two cohort studies. Multivariable-adjusted beta-binomial models estimated the odds of tumor T cell positivity by contraceptive history.

**Results:**

High-grade serous tumors had the highest percentage of tumor cells positive for total T cells (CD3^+^ mean=3.4%, SD = 6.1) and each T cell subtype. Ever (vs. never) IUD use was modestly associated with increased cytotoxic T cell infiltration (CD3^+^CD8^+^ OR:1.14, 95% CI:0.99–1.32), which was stronger among those with a history of endometriosis, postmenopausal women, and smokers. Conversely, OC use ≥1 year (vs. never) was associated with lower cytotoxic T cell odds (CD3^+^CD8^+^ OR:0.89, 95% CI:0.79–1.00; *p*-het=0.008). Increased odds of terminal T cell exhaustion were observed for IUD use only (CD3^+^PD1^+^TIM3^+^ OR:1.53, 95% CI:0.99–2.36), which was stronger among those who had ever used genital powder or BMI > 25 kg/m^2^.

**Conclusions:**

Pre-diagnostic contraception use may influence ovarian tumor immunity and may modulate cancer susceptibility.

## Introduction

Although oral contraception (OC) use reduces the risk of ovarian cancer by an estimated 15–30% [1, 2], use has declined over the last 20 years in favor of long-acting contraception methods, particularly intrauterine devices (IUDs) [3]. In a meta-analysis of IUD use and ovarian cancer risk, IUDs were associated with a reduced risk similar to OCs [4], although there was substantial heterogeneity across studies, ranging from 59% lower [5, 6] to 65% elevated risk [7]. Little is known about the potential biologic mechanisms linking IUD use with ovarian cancer risk.

Non-hormonal IUDs exert contraceptive effects through a localized inflammatory foreign body reaction [8], which includes the influx of immune cells. Consequently, the hypothesized mechanisms for the effect of non-hormonal IUDs on gynecologic cancer risk include attracting immune cells that clear premalignant cells, reducing menstrual flow, or changing sensitivity to steroid receptors through mediation by copper ions [9–12]. However, IUD use could enhance cancer development through increased inflammation, which plays a role in ovarian carcinogenesis [13–16]. Therefore, evaluating the ovarian tumor immune microenvironment may help clarify how IUD use interacts with the anti-tumor immune response. Here, we evaluated the use of IUDs or OCs in relation to T-cell infiltration in ovarian tumors among women from six epidemiologic studies.

## Materials and methods

### Study population and ovarian cancer ascertainment

This analysis included women with epithelial ovarian cancer from six studies: African American Cancer Epidemiology Study (AACES), Diseases of the Ovary and their Evaluation Study (DOVE), Hormones and Ovarian Cancer Prediction Study (HOPE), New England Case Control Study (NECC), Nurses’ Health Study (NHS), and NHSII. All participants provided written consent or implied consent by completing questionnaires. Protocols at each study site were approved by the relevant Institution Review Boards (IRBs) or ethics committee and those of relevant registries.

AACES (Phase 1) is a multi-center population-based case–control study that enrolled 592 women aged 20 to 79 with newly diagnosed epithelial ovarian cancer between 2010 and 2015 from geographic regions within the US with a high proportion of African American individuals [17]. Ovarian cancer cases were identified through rapid case ascertainment involving gynecologic oncology departments at individual hospitals and cancer registries [18]. Participants completed computer-assisted telephone interviews. Data were collected and managed at Duke University, the University of Virginia, and Emory University, where IRB approval was obtained.

DOVE is a population-based ovarian cancer case-control study that enrolled 1502 ovarian cancer cases ages 35–74 years from 13 counties in Western Washington State between 2002 and 2009 [19]. Ovarian cancer cases were identified using a population-based cancer registry that participated in the SEER Registry Program [18]. Participants completed in-person interviews. The IRB of the Fred Hutchinson Cancer Research Center approved the study protocol.

The HOPE study enrolled women with primary epithelial ovarian, fallopian tube, or peritoneal cancer between 2003 and 2008 who were residents of the contiguous region consisting of Western Pennsylvania, Eastern Ohio, and Western New York State [20]. Cases were identified from hospital tumor registries, clinical practices, and pathology databases. Participants completed interviews at home with trained interviewers. The study protocol was approved by the University of Pittsburgh IRB and human subjects committees representing each hospital where cases were identified.

NECC is a population-based ovarian cancer case-control study that enrolled 2203 women with ovarian cancer aged 18–80 years residing in Eastern Massachusetts and New Hampshire between 1992 and 2008 [21]. Ovarian cancer cases were identified using hospital records and state cancer registries. All participants completed in-person interviews. The IRB of Brigham and Women’s Hospital and Dartmouth Medical School approved the study protocol.

NHS is a prospective cohort study established in 1976 that enrolled 121,700 female registered nurses, 30–55 years old, residing in 11 US states who completed and returned a mailed questionnaire [22]. NHSII began in 1989 and enrolled 116,429 female registered nurses, aged 25 to 42 years, from 14 US states who completed a similar questionnaire [23]. The updated exposure status and disease outcomes were assessed using biennial questionnaires. Incident ovarian cancer cases were identified using self-report, reports from family, or linkage with the National Death Index [24]. Ovarian cancer diagnoses were confirmed by a medical record review or linkage to state cancer registries. The study protocol was approved by the Institutional Review Boards of the Brigham and Women’s Hospital and the Harvard T.H. Chan School of Public Health, and those of participating registries as required.

### Contraception use and covariates

During interviews, AACES participants were asked to report what birth control method(s) they had used, along with age at first use, the number of episodes, and duration of use. Age at last use or time since last use was not assessed. HOPE participants used life calendars to recall contraception use, including type, frequency, duration, and reason. In DOVE and NECC, participants reported the method and name of contraception as well as the calendar date of first and last use or age of first use and duration. For DOVE, HOPE, and NECC participants, duration, age at first, age at last, and time since last contraception use were determined using start and stop dates. NHS/NHSII participants reported current and previous contraception use at baseline and in biennial questionnaires. Self-reported duration of OC use was assessed among NHS participants, while NHSII participants provided age at first and last contraception use, which was used to determine duration and time since last use. Use status was abstracted from the most recently completed questionnaire prior to ovarian cancer diagnosis with contraceptive information. IUD and OC use were coded as having ever/never used each method of contraception, while duration, age at first use, time since last use, and order of first use and first birth among parous women for each method of contraception were dichotomized at the mean for stratified analyses. Although most studies did not specify the type of IUD used, the timing of questionnaire completion suggests that the majority of participants would have used contraception before hormonal IUDs became available in the US. Therefore, IUD use in our study likely refers to inert or non-hormonal IUDs.

Covariates included age at ovarian cancer diagnosis, self-reported race (white or presumed white, black or African American, Asian, Hispanic and other), parity (never pregnant, 1 full term birth, 2+ full term births), prior tubal ligation (yes, no/unknown), genital powder use (ever, never), history of endometriosis (yes, no, missing/unknown), family history of breast or ovarian cancer (yes, no/unknown), menopausal status at diagnosis (pre-, post-, missing/unknown). Tumor histotype was based on pathologist H&E slide review (high-grade serous, low-grade serous, mucinous, endometrioid, clear cell, other or unknown epithelial).

### Measurement of tumor infiltrating immune markers

**Fig. 1:**
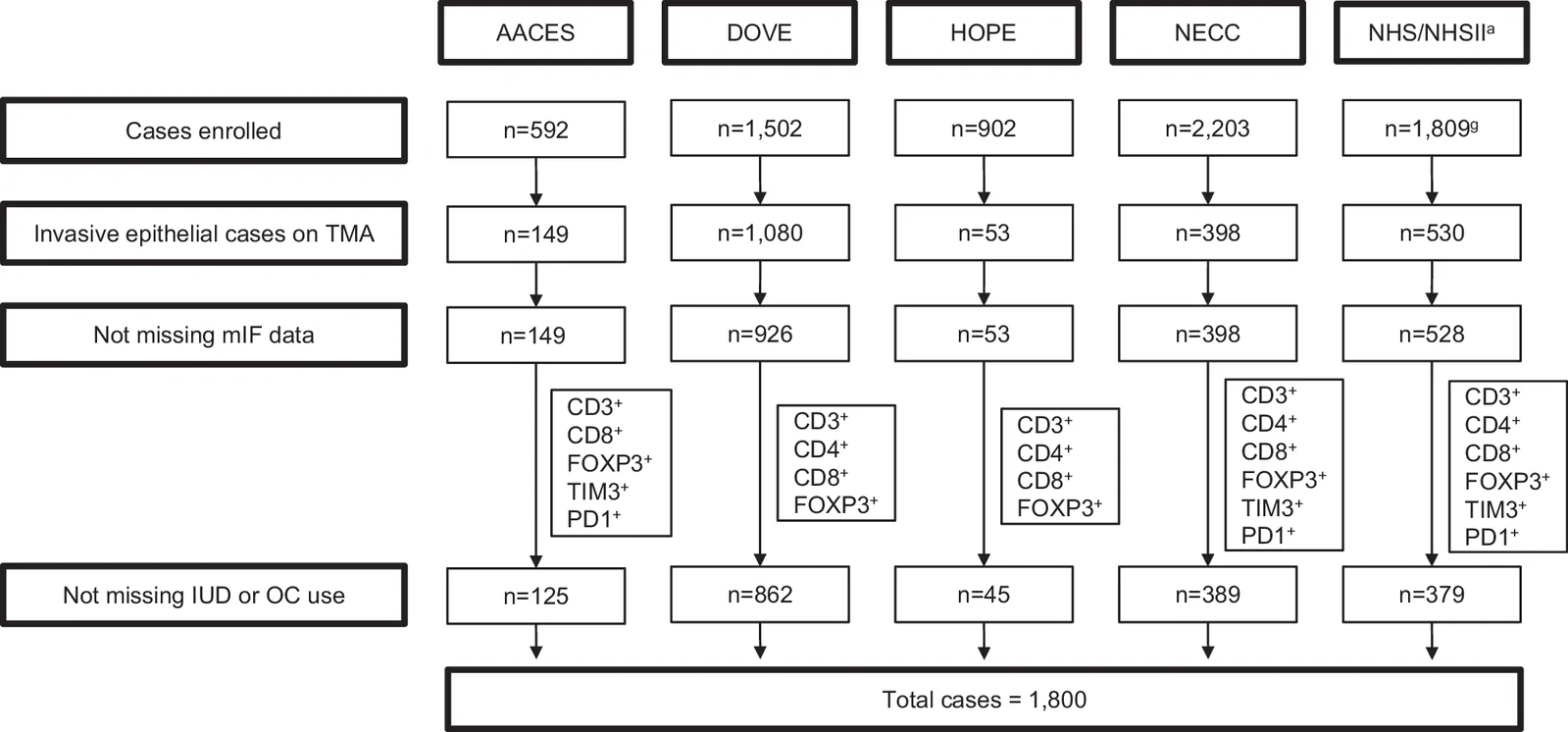
Overview of cases included in analysis by study site. ^a^ Schildkraut JM, Johnson C, Dempsey LF, Qin B, Terry P, Akonde M, et al. Survival of epithelial ovarian cancer in Black women: a society to cell approach in the African American cancer epidemiology study (AACES). Cancer Causes Control. 2023;34(3):251-65. ^b^ Peres LC, Cushing-Haugen KL, Anglesio M, Wicklund K, Bentley R, Berchuck A, et al. Histotype classification of ovarian carcinoma: A comparison of approaches. Gynecol Oncol. 2018;151(1):53-60. ^c^ Goode EL, Block MS, Kalli KR, Vierkant RA, Chen W, Fogarty ZC, et al. Dose-Response Association of CD8+ Tumor-Infiltrating Lymphocytes and Survival Time in High-Grade Serous Ovarian Cancer. JAMA oncology. 2017;3(12):e173290. ^d^ Shafrir AL, Rice MS, Gupta M, Terry KL, Rosner BA, Tamimi RM, et al. The association between reproductive and hormonal factors and ovarian cancer by estrogen-α and progesterone receptor status. Gynecol Oncol. 2016;143(3):628-35. ^e^ Harris HR, Rice MS, Shafrir AL, Poole EM, Gupta M, Hecht JL, et al. Lifestyle and Reproductive Factors and Ovarian Cancer Risk by p53 and MAPK Expression. Cancer Epidemiol Biomarkers Prev. 2018;27(1):96-102. ^f^ Hathaway CA, Conejo-Garcia JR, Fridley BL, Rosner B, Saeed-Vafa D, Moran Segura C, et al. Measurement of Ovarian Tumor Immune Profiles by Multiplex Immunohistochemistry: Implications for Epidemiologic Studies. Cancer Epidemiol Biomarkers Prev. 2023;32(6):848-53. AACES African-American Cancer Epidemiology Study, DOVE Diseases of the Ovary and their Evaluation, HOPE Hormones and Ovarian Cancer Prediction Study, mIF multiplex immunofluorescence, NECC New England Case Control Study, NHS Nurses’ Health Study. ^f^Number of incident epithelial ovarian cancer cases as of 2020.

Formalin-fixed paraffin-embedded treatment-naïve ovarian tumor samples were identified and obtained from consenting participants. There were 149 cases from AACES who provided tumor samples for tissue microarrays (TMAs), 1080 from DOVE, 53 from HOPE, 398 from NECC, and 530 from NHS/NHSII. All samples were reviewed by gynecologic pathologists who recorded information on tumor characteristics (e.g., morphology, grade, histology). There were 24 TMAs (2 in AACES, 8 in DOVE, 1 in HOPE, 6 in NECC, and 7 in NHS/NHSII). TMAs were constructed using four 0.6 mm (DOVE), up to three 1 mm (NECC), or three 0.6 mm (AACES, HOPE, NECC, NHS/NHSII) cores per case. The process of collecting ovarian tumor tissue for TMAs has been previously described in AACES [25], DOVE [26], HOPE [27], NECC [28, 29], and NHS/NHSII [7, 28, 29] (Fig. 1).

Using multiplex immunofluorescence (mIF), TMA slides were stained and analyzed for CD3^+^ (total T cells), CD3^+^CD8^+^ (cytotoxic), and CD3^+^CD4^+^FOXP3^+^ (CD3^+^FOXP3^+^ in AACES, regulatory) T cell markers in all TMAs. CD3^+^CD4^+^ (helper) T cells were measured in DOVE, HOPE, NECC, and NHS/NHSII TMAs (Supplementary Table 1). An additional panel of T cell exhaustion markers, including CD3^+^TIM3^+^ (likely exhausted) and CD3^+^PD1^+^TIM3^+^ (terminally exhausted), was measured in AACES, NECC, and NHS/NHSII TMAs. All mIF assays were completed at the Moffitt Cancer Center using the Akoya Biosciences Opal^TM^ 7-Color Automation immunohistochemistry kit and the Vectra®3 Automated Quantitative Pathology Imaging System. Use of the Opal panel allowed for staining of five immunohistochemistry antibodies at once, in addition to 4′,6-diamidino-2-phenylindole (DAPI) and pan-cytokeratin using a tyramide-signal kit for amplification. Slide images were captured via multispectral Vectra® microscope, and specialized image analysis and data consolidation were performed using HALO software (Indica Labs, New Mexico). A random forest machine learning algorithm was used to define epithelial and stroma compartments based on DAPI and pan-cytokeratin staining [7]. T cell subpopulations were then defined by co-expression of markers, e.g., T-helper (CD3^+^CD4^+^). Tumor epithelium and stroma were scored separately [30]. For each cell, a positivity threshold within the nucleus or cytoplasm was determined for each marker according to the visual intensity [7]. Cells were considered positive for the marker of interest if the fluorescence intensity for all relevant stains exceeded the established positivity threshold.

### Statistical analysis

Of the 2210 invasive epithelial ovarian cancer cases with tumor tissue, there were 2054 cases included in the TMAs with mIF data. Cases were excluded if IUD or OC use prior to diagnosis was unknown (*n* = 254), resulting in 1800 epithelial ovarian cancer cases in the analysis. For each T cell type, we assessed the mean percent of tumor cells positive for that marker and standard deviation (SD). Similar to previous analyses of ovarian tumor T cells [31, 32], beta-binomial models with a random effect accounting for multiple cores per tumor were applied to estimate odds ratios (OR) and 95% confidence intervals (CIs), using the positive number of cells for each cell type out of the total number of cells. The ORs can be interpreted as the ratio of odds that a tumor epithelium cell was positive for the immune cell type of interest, comparing ever use of IUD or OC use for at least one year to those who had never used the respective method of contraception [33].

Models for T cell subtypes (helper, cytotoxic, regulatory, exhausted) were adjusted for the tertile of total parent cell (CD3^+^) as a fixed effect to account for the high correlation between total T cells and T cell subtypes. All models were adjusted for age at ovarian cancer diagnosis, parity, tubal ligation, family history of breast or ovarian cancer in first-degree relative, study site (AACES, DOVE, HOPE, NECC, NHS, NHSII), histotype, and mutually adjusted for use of the other contraception method (ever, never). Results were reported for all histotypes combined. Associations of ever IUD or OC use with T cells were first determined within each study, and Cochrane’s Q statistic for random-effects meta-analysis to assess potential heterogeneity by study site was calculated (Supplementary Table 2). Given the lack of heterogeneity, data were pooled for analyses and the Wald test for equality of coefficients to determine whether the association of IUD use with each marker was significantly different from the association of OC. As inflammation is one of the hypothesized pathways through which the ovarian tumor immune microenvironment may be affected by contraception use, we conducte exploratory stratified analyses by potential contributors to inflammation (BMI, menopausal status, genital powder use, endometriosis, smoking history) and assessed the interaction term of either IUD or OC use with each potential modifier for statistical significance. We also examined duration and timing of contraception use, including age at first use, time since last use, and order of contraception use relative to first birth among parous women, compared with never use. Sensitivity analyses included assessment of exclusive use of IUD or OC, comparison of ever use of IUD use to OC use for at least five years, and histotype-specific estimates where sample size was sufficient. All hypothesis tests were two-sided, with a Bonferroni-corrected significance threshold of *α* = 0.008 to adjust for multiple comparisons across the six T cell subtypes. Analyses were conducted using SAS 9.4 (SAS Institute Inc.) and the statistical computing language R, version 4.2.0.

## Results

A total of 1800 participants from AACES, DOVE, HOPE, NECC, NHS, and NHSII with epithelial ovarian tumors and a known history of IUD and OC use were included. Age at ovarian cancer diagnosis was similar among ever and never IUD users (57.0±7.3, 58.0±10.6 years, respectively), but those who had ever used OCs were slightly younger at diagnosis (55.4±8.9 years) compared to never users (60.5±10.7) (Table 1). Within this population, 313 women (17.4%) reported having ever used an IUD and 923 (51.3%) reported having used OC for at least one year. There was no difference in the history of endometriosis by IUD use (*n* = 31, 9.9% ever user vs. *n* = 140, 9.4% never use), while those who had ever used OCs were more likely to have a history of endometriosis (*n* = 106, 11.5% ever user vs. *n* = 66, 7.5% never use). Genital powder use was lower among those who had ever used an IUD (*n* = 59, 18.9%) versus never (*n* = 338, 22.7%), as well as those who had ever (*n* = 168, 18.2%) versus never used OCs (*n* = 229, 26.1%). Those who had ever used an IUD were more likely to have high-grade serous tumors (*n* = 234, 74.8%) compared to never users (*n* = 978, 65.8%), while there were no differences between OC users (*n* = 620, 67.2%) and never users (n = 592, 67.5%).

**Table 1:** Population characteristics of AACES, DOVE, HOPE, NECC, NHS, and NHSII ovarian cancer cases by intrauterine device (IUD) and oral contraception (OC) use (*n* = 1800)^a^.

	Ever IUD	Never IUD	Ever OC ≥1 year	Never or OC <1 year
**Total**	313 (17.4)	1487 (82.6)	923 (51.3)	877 (48.7)
Age at diagnosis^b^, years	57.0 ± 7.3	58.0 ± 10.6	55.4 ± 8.9	60.5 ± 10.7
Year of diagnosis^b^	2005 ± 4.1	2004 ± 6.0	2005 ± 4.7	2003 ± 6.5
Ever used IUD	313 (100.0)	0 (0.0)	192 (20.8)	121 (13.8)
Duration of IUD use, years^b, c^	4.5 ± 5.4	–	3.0 ± 4.9	4.3 ± 6.2
Age at first IUD use, years^b, c^	25.7 ± 5.0	–	26.0 ± 4.5	25.2 ± 5.8
Time since last IUD use, years^b, c^	27.4 ± 8.3	-	27.4 ± 7.3	27.3 ± 9.8
Ever used OC, at least 1 year	192 (61.3)	731 (49.2)	923 (100.0)	0 (0.0)
Duration of oral contraception use, years				
Never	70 (22.4)	580 (39.0)	0 (0.0)	650 (74.1)
<1	51 (16.3)	176 (11.8)	0 (0.0)	227 (25.9)
1 to <5	113 (36.1)	370 (24.9)	482 (52.2)	0 (0.0)
5 to <10	59 (18.9)	221 (14.9)	281 (30.4)	0 (0.0)
10+	20 (6.4)	140 (9.4)	160 (17.3)	0 (0.0)
Duration of OC use, years^b, d^	3.0 ± 3.7	3.1 ± 4.8	6.0 ± 4.9	0.1 ± 0.2
Age at first OC use, years^b, d^	21.1 ± 4.9	22.1 ± 5.2	21.3 ± 4.7	23.9 ± 6.4
Time since last OC use, years^b, d^	29.5 ± 8.8	25.9 ± 10.9	25.8 ± 10.4	30.7 ± 10.6
Family history of breast or ovarian cancer^e^				
Yes	68 (21.7)	299 (20.1)	207 (22.4)	160 (18.2)
No or not reported	245 (78.3)	1188 (79.9)	716 (77.6)	717 (81.8)
History of endometriosis				
Yes	31 (9.9)	140 (9.4)	106 (11.5)	66 (7.5)
No	264 (84.4)	1063 (71.5)	743 (80.5)	583 (66.5)
Missing or unknown	18 (5.8)	284 (19.1)	74 (8.0)	228 (26.0)
Genital powder use (ever)				
Yes	59 (18.9)	338 (22.7)	168 (18.2)	229 (26.1)
No	153 (48.9)	756 (50.8)	445 (48.2)	464 (52.9)
Missing or unknown	101 (32.3)	393 (26.4)	310 (33.6)	184 (21.0)
Number of full term births^f^				
0	22 (7.0)	296 (19.9)	150 (16.3)	167 (19.0)
1	50 (16.0)	208 (14.0)	160 (17.3)	99 (11.3)
2	131 (41.9)	478 (32.2)	363 (39.3)	246 (28.1)
3	72 (23.0)	280 (18.8)	172 (18.6)	181 (20.6)
4 or more	38 (12.1)	225 (15.1)	79 (8.5)	184 (21.0)
Menopause status				
Premenopausal	68 (21.7)	394 (26.5)	278 (30.1)	185 (21.1)
Postmenopausal	220 (70.3)	940 (63.2)	544 (58.9)	615 (70.1)
Missing or unknown	25 (8.0)	153 (10.3)	101 (10.9)	77 (8.8)
Race/ethnicity				
White or presumed white	285 (91.1)	1300 (87.4)	823 (89.2)	762 (86.9)
Black or African American	16 (5.1)	129 (8.7)	72 (7.8)	73 (8.3)
Asian	5 (1.6)	38 (2.6)	16 (1.7)	27 (3.1)
Hispanic, Native American, and other	7 (2.2)	20 (1.3)	12 (1.3)	15 (1.7)
Body mass index, kg/m^2^				
<25	150 (47.9)	661 (44.5)	418 (45.3)	393 (44.8)
25^+^	164 (52.1)	778 (52.3)	490 (53.1)	451 (51.4)
Missing or unknown	0 (0.0)	48 (3.2)	15 (1.6)	33 (3.8)
Smoking status				
Ever smoked	168 (53.7)	685 (46.1)	453 (49.1)	399 (45.5)
Never smoked or did not report smoking	145 (46.3)	802 (53.9)	470 (50.9)	478 (54.5)
Site				
AACES	14 (4.5)	111 (7.5)	65 (7.0)	60 (6.8)
DOVE	208 (66.5)	655 (44.0)	536 (58.1)	326 (37.2)
HOPE	9 (2.9)	36 (2.4)	23 (2.5)	22 (2.5)
NECC	65 (20.8)	324 (21.8)	170 (18.4)	219 (25.0)
NHS	15 (4.8)	277 (18.6)	68 (7.4)	224 (25.5)
NHSII	2 (0.6)	85 (5.7)	61 (6.6)	26 (3.0)
Tumor histotype				
High-grade serous	234 (74.8)	978 (65.8)	620 (67.2)	592 (67.5)
Low-grade serous	4 (1.3)	39 (2.6)	23 (2.5)	20 (2.3)
Mucinous	10 (3.2)	63 (4.2)	32 (3.5)	41 (4.7)
Endometrioid	40 (12.8)	250 (16.8)	156 (16.9)	134 (15.3)
Clear cell	15 (4.8)	109 (7.3)	63 (6.8)	61 (7.0)
Other	10 (3.2)	48 (3.2)	29 (3.1)	29 (3.3)

*AACES* African-American Cancer Epidemiology Study, *DOVE* Diseases of the Ovary and their Evaluation, *HOPE* Hormones and Ovarian Cancer Prediction Study, *NECC* New England Case Control Study, *NHS* Nurses’ Health Study

^a^Expressed as *n*(%) unless otherwise stated.

^b^Mean ± SD.

^c^Among IUD users.

^d^Among OC users.

^e^Self-reported family history of ovarian cancer in mother and/or sister(s).

^f^Self-reported pregnancies carried to 20 weeks or greater.

We reported the mean number of tumor cells per core as well as the mean percent of cells positive for each tumor infiltrate, overall and by histotype, in Table 2. Overall, endometrioid tumors had the highest percentage tumor cells per core (mean=75.2%, SD: 24.3), which was similar to high grade serous tumors (mean=74.7%, SD: 23.9), followed by low grade serous (mean=68.5%, SD: 24.0), clear cell (mean=67.8%, SD: 25.6), and mucinous tumors (mean=60.1%, SD: 27.8). High grade serous tumors had the highest percentage of tumor cells positive for total T cells (CD3^+^ mean=3.4%, SD = 6.1) and had the highest percent of tumor cells positive for each T cell subtype, however, 1% of tumor cells or less were positive for most markers.

**Table 2:** Mean percent of cells positive for tumor immune infiltrates and standard deviation (SD) per core among AACES, DOVE, HOPE, NECC, NHS, and NHS2 participants by histological subtype.

		Histological subtype^a^				
	All histotypes *n* = 1800	High-grade serous *n* = 1212	Low-grade serous *n* = 43	Mucinous *n* = 73	Endometrioid *n* = 290	Clear cell *n* = 124
Tumor cells^b^	72.7 ± 25.4	74.7 ± 23.9	68.5 ± 24.0	60.1 ± 27.8	75.2 ± 24.3	67.8 ± 25.6
CD3^+^ (total)	2.9 ± 5.6	3.4 ± 6.1	1.8 ± 2.5	1.4 ± 3.9	2.4 ± 5.1	1.2 ± 2.2
CD3^+^CD4^+^ (helper)^c^	0.7 ± 2.3	0.9 ± 2.5	0.4 ± 0.9	0.2 ± 0.9	0.5 ± 1.4	0.2 ± 0.5
CD3^+^CD8^+^ (cytotoxic)	0.9 ± 2.3	1.0 ± 2.4	0.5 ± 1.0	0.4 ± 1.5	0.8 ± 2.2	0.4 ± 0.9
CD3^+^CD4^+^FOXP3^+^ (regulatory)	0.2 ± 0.7	0.2 ± 0.8	0.2 ± 0.5	0.1 ± 0.6	0.08 ± 0.3	0.03 ± 0.1
CD3^+^TIM3^+^ (exhausted)^d^	1.0 ± 3.5	1.2 ± 3.9	0.8 ± 2.0	0.06 ± 0.1	0.8 ± 2.6	0.4 ± 1.1
CD3^+^PD1^+^TIM3^+^ (terminally exhausted)^d^	0.4 ± 1.6	0.5 ± 1.7	0.3 ± 1.1	0.02 ± 0.06	0.4 ± 1.8	0.2 ± 0.5

*CI* confidence interval, *AACES* African-American Cancer Epidemiology Study, *DOVE* Diseases of the Ovary and their Evaluation, *HOPE* Hormones and Ovarian Cancer Prediction Study, *IQR* interquartile range, *NECC* New England Case Control Study, *NHS* Nurses’ Health Study, *SD* standard deviation.

^a^Excludes unknown histological subtype (*n* = 58).

^b^Percent of total cells (includes tumor and stroma cells) that are classified as tumor.

^c^Excludes AACES.

^d^Excludes DOVE and HOPE.

While there was no significant association with total T cells (CD3^+^), having ever versus never used an IUD was associated with higher odds of cytotoxic T cell infiltration (CD3^+^CD8^+^ OR:1.14, 95% CI:0.99–1.32; Table 3). Additionally, there were increased odds of terminally exhausted T cells among IUD users versus non-users (CD3^+^PD1^+^TIM3^+^ OR:1.53, 95% CI:0.99–2.36). When assessing tumor T cell infiltration associated with use of IUD only compared to those who never used either IUD or OC, there was no longer an association of IUD use with CD3^+^CD8^+^ cytotoxic T cells (Supplementary Table 3). There were greater odds of both T cell exhaustion (CD3^+^TIM3^+^ OR: 1.86, 95% CI: 1.15–3.00) and terminal T cell exhaustion (C3^+^PD1^+^TIM3^+^ OR = 2.19, 95% CI: 1.20–4.02) among exclusive IUD users compared to those who had never used IUD or OC. Use of OC for at least one year versus never or less than one year of OC use and no IUD use was associated with lower odds of cytotoxic T cell infiltration (CD3^+^CD8^+^ OR:0.89, 95% CI: 0.79–1.00) (Table 3), which was nearly unchanged when assessing tumor T cell infiltration among those who exclusively used an OC with no history of IUD use (Supplementary Table 3; CD3^+^CD8^+^ OR: 0.86, 95% CI: 0.75–0.97). We did not have an adequate sample size to evaluate the associations of IUD use with tumor immune markers by all histotypes, but did observe similar results when restricting to high-grade serous or endometrioid tumors (Supplementary Table 4).

**Table 3:** Odds ratios (OR) and 95% confidence intervals (CI) for positivity of tumor immune infiltrations associated with ever use of intrauterine device (IUD) or oral contraception (OC) among AACES, DOVE, HOPE, NECC, NHS, and NHSII participants (*n* = 1800)^a^.

T cell type	Ever used IUD vs. never	Ever used OC ≥1 year vs. never	*p*-value^e^
CD3^+^ (total)	1.00 (0.85–1.18)	0.97 (0.85–1.10)	0.78
CD3^+^CD4^+b,c^ (helper)	1.06 (0.91–1.25)	1.07 (0.94–1.21)	0.99
CD3^+^CD8^+b^ (cytotoxic)	1.14 (0.99–1.32)	0.89 (0.79–1.00)	0.008
CD3^+^CD4^+^FOXP3^+b^ (regulatory)	0.98 (0.81–1.17)	1.06 (0.92–1.23)	0.48
CD3^+^TIM3^+b,d^ (exhausted)	1.30 (0.92–1.82)	0.90 (0.72–1.11)	0.07
CD3^+^PD1^+^TIM3^+b,d^ (terminally exhausted)	1.53 (0.99–2.36)	0.92 (0.70–1.22)	0.05

*CI* confidence interval, *AACES* African-American Cancer Epidemiology Study, *DOVE* Diseases of the Ovary and their Evaluation, *HOPE* Hormones and Ovarian Cancer Prediction Study, *NECC* New England Case Control Study, *NHS* Nurses’ Health Study.

^a^ORs were calculated using beta-binomial models using the positive number of cells for each cell type out of the total number of cells adjusting for age at cancer diagnosis, parity (never pregnant, 1 full term birth, 2 or more full term births), family history of breast or ovarian cancer in first degree relative (yes, no or not reported) histotype (high grade serous, low grade serous, mucinous, endometrioid, clear cell, other), study site (AACES, DOVE, HOPE, NECC, NHS, NHSII), and mutually adjusted for use of contraception method (never, ever).

^b^Additionally adjusted for tertile of total T cells (CD3^+^).

^c^Excludes AACES.

^d^Excludes DOVE and HOPE.

^e^Wald test for equality of coefficients.

We assessed potential effect modification by other contributors of inflammation, including history of endometriosis, genital powder use, menopausal status, BMI, and smoking status (Table 4). There were no significant differences in T cell infiltration associated with IUD use by endometriosis status; however, there were significantly higher odds of cytotoxic T cells by IUD use among those with endometriosis (CD3^+^CD8^+^ OR: 1.66, 95% CI: 1.12–2.47) that was not observed among those without (OR:1.08, 95% CI: 0.92–1.26). The interaction between IUD use and endometriosis was not statistically significant. There was some suggestion that those who had ever used an IUD and genital power had greater overall (CD3^+^TIM3^+^ OR: 1.57, 95% CI: 0.94–2.63) and terminal T cell exhaustion (CD3^+^PD1^+^TIM3^+^ OR: 2.45, 95% CI: 1.22–4.90) compared to those had never used genital power (OR: 1.07, 95% CI: 0.69–1.67; OR: 1.16, 95% CI: 0.67–2.00; respectively), although there was no significant joint interaction between IUD and genital powder use. IUD users who were postmenopausal at the time of diagnosis had significantly higher odds of cytotoxic T cells (CD3^+^CD8^+^ OR: 1.20, 95% CI: 1.00–2.42), while there was no association with IUD use among those who were premenopausal at the time of diagnosis (OR: 1.11, 95% CI: 0.83–1.49). When assessing OC use, there was no association of OC use with T cells among postmenopausal women. However, OC users who were premenopausal at the time of diagnosis had significantly lower odds of T cell exhaustion (CD3^+^TIM3^+^ OR: 0.59, 95% CI: 0.36–0.98) that was not observed among postmenopausal women (OR: 1.04, 95% CI: 0.79–1.37). Among those who had ever used an IUD with a BMI over 25 kg/m^2^, greater cytotoxic T cell infiltration (CD3^+^CD8^+^ OR: 1.20, 95% CI: 0.98–1.48) and terminal T cell exhaustion was observed (CD3^+^PD1^+^TIM3^+^ OR: 2.10, 95% CI: 1.17–3.75) while there was no association among those with a BMI less than 25 kg/m^2^ (OR: 1.08, 95% CI: 0.88–1.32; OR: 1.15, 95% CI: 0.63–2.11; respectively), and no significant joint interaction. IUD users who had ever smoked were also more likely to have increasing odds of cytotoxic T cell infiltration (CD3^+^CD8^+^ OR: 1.26, 95% CI: 1.03–1.53), while there was no association of IUD use among non-smokers (OR: 1.06, 95% CI: 0.86–1.31). This interaction did not reach statistical significance.

**Table 4:** Odds ratios (OR) and 95% confidence intervals (CI) for positivity of tumor immune infiltrations of T cells associated with contraception use stratified by potential contributors of inflammation among AACES, DOVE, HOPE, NECC, NHS, and NHSII participants (*n* = 1800)^a^.

	Ever used IUD vs. never		Ever used OC ≥1 year vs. never	
**T cell type**	**Endometriosis**^e,f^			
	**Yes, history of endometriosis**	**No history of endometriosis**	**Yes, history of endometriosis**	**No history of endometriosis**
CD3^+^ (total)	1.55 (0.94–2.55)	0.95 (0.80–1.14)	1.14 (0.77–1.70)	0.95 (0.80–1.14)
CD3^+^CD4^+b,c^ (helper)	1.20 (0.82–1.75)	1.07 (0.90–1.27)	0.94 (0.67–1.30)	1.01 (0.87–1.17)
CD3^+^CD8^+b^ (cytotoxic)	1.66 (1.12–2.47)	1.08 (0.92–1.26)	1.06 (0.76–1.48)	0.94 (0.82–1.07)
CD3^+^CD4^+^FOXP3^+b,c^ (regulatory)	1.27 (0.86–1.88)	0.95 (0.78–1.16)	0.76 (0.53–1.07)	1.09 (0.93–1.29)
CD3^+^TIM3^+b,d^ (exhausted)	1.26 (0.76–2.11)	1.20 (0.81–1.77)	0.86 (0.56–1.31)	0.92 (0.71–1.21)
CD3^+^PD1^+^TIM3^+b,d^ (terminally exhausted)	–	1.33 (0.81–2.29)	–	0.97 (0.69–1.37)
	**Genital powder use**^f^			
**T cell type**	**Yes, ever used**	**No, never used**	**Yes, ever used**	**No, never used**
CD3^+^ (total)	0.89 (0.63–1.27)	1.02 (0.82–1.28)	0.99 (0.76–1.29)	0.97 (0.81–1.15)
CD3^+^CD4^+b,c^ (helper)	1.07 (0.73–1.57)	1.13 (0.89–1.43)	0.97 (0.72–1.32)	1.08 (0.90–1.31)
CD3^+^CD8^+b^ (cytotoxic)	1.20 (0.86–1.67)	1.14 (0.93–1.39)	0.82 (0.63–1.06)	0.87 (0.75–1.01)
CD3^+^CD4^+^FOXP3^+b,c^ (regulatory)	0.99 (0.66–1.48)	0.93 (0.70–1.24)	1.03 (0.76–1.39)	1.18 (0.95–1.47)
CD3^+^TIM3^+b,d^ (exhausted)	1.57 (0.94–2.63)	1.07 (0.69–1.67)	1.12 (0.79–1.61)	0.78 (0.59–1.03)
CD3^+^PD1^+^TIM3^+b,d^ (terminally exhausted)	2.45 (1.22–4.90)	1.16 (0.67–2.00)	1.19 (0.73–1.95)	0.82 (0.58–1.15)
	**Menopausal status**^c,f^			
**T cell type**	**Postmenopausal**	**Premenopausal**	**Postmenopausal**	**Premenopausal**
CD3^+^ (total)	1.07 (0.87–1.30)	1.11 (0.78–1.58)	0.92 (0.78–1.09)	1.13 (0.88–1.46)
CD3^+^CD4^+b,c^ (helper)	1.13 (0.93–1.36)	1.16 (0.85–1.60)	1.15 (0.98–1.35)	0.93 (0.74–1.18)
CD3^+^CD8^+b^ (cytotoxic)	1.20 (1.00–1.42)	1.11 (0.83–1.49)	0.90 (0.78–1.04)	0.94 (0.76–1.17)
CD3^+^CD4^+^FOXP3^+b,c^ (regulatory)	1.07 (0.85–1.34)	0.97 (0.63–1.48)	1.09 (0.90–1.31)	0.92 (0.68–1.26)
CD3^+^TIM3^+b,d^ (exhausted)	1.49 (0.97–2.28)	1.14 (0.58–2.25)	1.04 (0.79–1.37)	0.59 (0.36–0.98)
CD3^+^PD1^+^TIM3^+b,d^ (terminally exhausted)	1.55 (0.89–2.70)	1.11 (0.45–2.70)	1.14 (0.80–1.64)	0.55 (0.29–1.06)
	**Body mass index**^f,g^			
**T cell type**	**BMI ≥25 kg/m**^2^	**BMI <25 kg/m**^2^	**BMI ≥25 kg/m**^2^	**BMI <25 kg/m**^2^
CD3^+^ (total)	1.21 (0.96–1.53)	0.80 (0.64–1.00)	1.00 (0.83–1.20)	0.94 (0.79–1.13)
CD3^+^CD4^+b,c^ (helper)	1.07 (0.85–1.33)	1.08 (0.86–1.35)	1.04 (0.87–1.26)	1.10 (0.92–1.33)
CD3^+^CD8^+b^ (cytotoxic)	1.20 (0.98–1.48)	1.08 (0.88–1.32)	0.91 (0.77–1.07)	0.92 (0.78–1.08)
CD3^+^CD4^+^FOXP3^+b,c^ (regulatory)	1.00 (0.77–1.29)	0.98 (0.75–1.29)	1.05 (0.85–1.29)	1.08 (0.87–1.34)
CD3^+^TIM3^+b,d^ (exhausted)	1.45 (0.91–2.31)	1.18 (0.74–1.88)	0.83 (0.62–1.12)	0.96 (0.71–1.32)
CD3^+^PD1^+^TIM3^+b,d^ (terminally exhausted)	2.10 (1.17–3.75)	1.15 (0.63–2.11)	0.81 (0.55–1.19)	1.08 (0.71–1.63)
	**Smoking status**^f^			
	**Yes, ever smoked**	**No, never smoked**	**Yes, ever smoked**	**No, never smoked**
CD3^+^ (total)	1.08 (0.87–1.35)	0.95 (0.75–1.21)	0.95 (0.79–1.15)	0.99 (0.82–1.18)
CD3^+^CD4^+b,c^ (helper)	1.18 (0.95–1.46)	0.95 (0.75–1.20)	1.07 (0.89–1.30)	1.08 (0.90–1.29)
CD3^+^CD8^+b^ (cytotoxic)	1.26 (1.03–1.53)	1.06 (0.86–1.31)	0.91 (0.77–1.07)	0.90 (0.76–1.05)
CD3^+^CD4^+^FOXP3^+b,c^ (regulatory)	1.11 (0.87–1.42)	0.87 (0.66–1.15)	1.08 (0.87–1.33)	1.08 (0.88–1.32)
CD3^+^TIM3^+b,d^ (exhausted)	1.53 (0.93–2.52)	1.20 (0.76–1.88)	0.93 (0.68–1.28)	0.90 (0.67–1.21)
CD3^+^PD1^+^TIM3^+b,d^ (terminally exhausted)	1.69 (0.89–3.18)	1.40 (0.78–2.50)	1.12 (0.74–1.68)	0.84 (0.58–1.24)

^a^ORs were calculated using beta-binomial models using the positive number of cells for each cell type out of the total number of cells adjusting for age at cancer diagnosis, parity (never pregnant, 1 full term birth, 2 or more full term births), family history of breast or ovarian cancer in first degree relative (yes, no or not reported), study site (AACES, DOVE, HOPE, NECC, NHS, NHSII), histotype (high grade serous, low grade serous, mucinous, endometrioid, clear cell, other), and mutually adjusted for use of contraception method (never, ever).

^b^Additionally adjusted for tertile of total T cells (CD3^+^).

^c^Excludes AACES.

^d^Excludes DOVE and HOPE.

^e^Excludes NHS participants who did not report history of endometriosis.

^f^*p*-value for interaction term of each potential contributor of inflammation with method of contraception was not statistically significant.

^g^Measured up to 5 years prior to diagnosis.

We also assessed differences in either IUD or OC use (among ever users of the respective contraception) when dichotomized by mean duration of use, age at first use, time since last use, among parous and nulliparous women, and order of first contraception use relative to first birth among parous women and compared to never users (Table 5). Parous women who had ever used an IUD had greater odds of cytotoxic T cell infiltration (CD3^+^CD8^+^ OR: 1.18, 95% CI: 1.01–1.37) compared to non-users, which was not observed among nulliparous women (OR: 0.80, 95% CI: 0.49–1.31) but were not significantly different after correction for multiple testing (*p* = 0.04). Findings also indicated greater T cell exhaustion in parous IUD users versus non-users (CD3^+^TIM3^+^ OR:1.45, 95% CI: 1.01–2.08; CD3^+^PD1^+^TIM3^+^: 1.76, 95% CI: 1.11–2.78) while there was no association among nulliparous women (OR: 0.43, 95% CI: 0.15–1.23; OR:0.60, 95% CI: 0.15–2.37; *p*-value = 0.11, 0.34, respectively).

**Table 5:** Odds ratios (OR) and 95% confidence intervals (CI) for positivity of tumor immune infiltrations of T cells associated with contraception by mean duration and timing of use among AACES, DOVE, HOPE, NECC, NHS, and NHSII participants (*n* = 1800)^a^.

	Ever used IUD vs. never			Ever used OC ≥1 year vs. never		
	**Duration of IUD use**^**f,g**^			**Duration of OC use**^**g**^		
	**<2 years**	**2+ years**	***p***-**value**^**e**^	**1 to <5 years**	**5+ years**	***p***-**value**^**e**^
CD3^+^ (total)	1.04 (0.81–1.34)	0.95 (0.77–1.17)	0.55	0.97 (0.83–1.14)	0.89 (0.76–1.04)	0.44
CD3^+^CD4^+b,c^ (helper)	1.13 (0.90–1.42)	1.01 (0.83–1.22)	0.44	0.98 (0.84–1.15)	1.04 (0.89–1.21)	0.63
CD3^+^CD8^+b^ (cytotoxic)	1.17 (0.94–1.46)	1.11 (0.93–1.34)	0.74	0.92 (0.80–1.06)	0.83 (0.72–0.96)	0.34
CD3^+^CD4^+^FOXP3^+b^ (regulatory)	1.09 (0.84–1.41)	0.88 (0.70–1.10)	0.23	0.97 (0.81–1.16)	1.04 (0.97–1.25)	0.57
CD3^+^TIM3^+b,d^ (exhausted)	1.19 (0.65–2.18)	1.27 (0.83–1.95)	0.86	0.94 (0.71–1.24)	0.88 (0.67–1.15)	0.74
CD3^+^PD1^+^TIM3^+b,de^ (terminally exhausted)	1.36 (0.65–2.88)	1.46 (0.85–2.52)	0.89	0.89 (0.62–1.27)	1.02 (0.72–1.44)	0.58
	**Age at first IUD use**^**f,g**^			**Age at first OC use**^**f,g**^		
	**<25 years**	**25+ years**	***p***-**value**^**e**^	**<25 years**	**25+ years**	***p***-**value**^**e**^
CD3^+^ (total)	1.03 (0.81–1.31)	0.95 (0.76–1.17)	0.58	0.94 (0.80–1.09)	0.98 (0.78–1.24)	0.73
CD3^+^CD4^+b,c^ (helper)	1.01 (0.81–1.27)	1.08 (0.88–1.31)	0.70	0.97 (0.84–1.12)	1.09 (0.88–1.35)	0.37
CD3^+^CD8^+b^ (cytotoxic)	1.19 (0.97–1.47)	1.10 (0.91–1.33)	0.56	0.95 (0.82–1.08)	0.93 (0.76–1.14)	0.90
CD3^+^CD4^+^FOXP3^+b^ (regulatory)	0.83 (0.64–1.08)	1.07 (0.85–1.35)	0.16	1.02 (0.86–1.20)	1.04 (0.81–1.33)	0.89
CD3^+^TIM3^+b,d^ (exhausted)	1.05 (0.61–1.79)	1.41 (0.89–2.23)	0.41	0.86 (0.65–1.14)	0.83 (0.57–1.25)	0.94
CD3^+^PD1^+^TIM3^+b,d^ (terminally exhausted)	1.14 (0.56–2.31)	1.66 (0.94–2.91)	0.42	0.93 (0.65–1.33)	0.93 (0.58–1.52)	0.98
	**Time since last IUD use**^**f,g**^			**Time since last OC use**^**f,g**^		
	**<28 years**	**28+ years**	***p***-**value**^**e**^	**<28 years**	**28+ years**	***p***-**value**^**e**^
CD3^+^ (total)	1.05 (0.80–1.38)	1.02 (0.79–1.32)	0.87	1.01 (0.85–1.21)	0.90 (0.76–1.07)	0.35
CD3^+^CD4^+b,c^ (helper)	0.96 (0.75–1.21)	1.05 (0.84–1.31)	0.57	0.99 (0.84–1.17)	1.01 (0.85–1.19)	0.91
CD3^+^CD8^+b^ (cytotoxic)	1.16 (0.91–1.46)	1.15 (0.92–1.44)	0.97	0.89 (0.76–1.04)	0.96 (0.82–1.12)	0.49
CD3^+^CD4^+^FOXP3^+b^ (regulatory)	0.91 (0.68–1.22)	0.98 (0.75–1.29)	0.71	1.05 (0.86–1.27)	1.02 (0.85–1.23)	0.87
CD3^+^TIM3^+b,d^ (exhausted)	-	-	-	0.91 (0.66–1.26)	0.81 (0.58–1.13)	0.61
CD3^+^PD1^+^TIM3^+b,d^ (terminally exhausted)	-	-	-	0.87 (0.58–1.31)	1.00 (0.66–1.52)	0.64
	**Parity**			**Parity**		
	**Ever parous**	**Nulliparous**	***p***-**value**^**i**^	**Ever parous**	**Nulliparous**	***p***-**value**^**i**^
CD3^+^ (total)	1.04 (0.88–1.23)	0.60 (0.34–1.08)	0.07	0.98 (0.85–1.13)	0.91 (0.68–1.21)	0.75
CD3^+^CD4^+b,c^ (helper)	1.07 (0.91–1.26)	1.06 (0.57–1.97)	0.81	1.12 (0.97–1.29)	0.89 (0.65–1.21)	0.44
CD3^+^CD8^+b^ (cytotoxic)	1.18 (1.01–1.37)	0.80 (0.49–1.31)	0.04	0.95 (0.83–1.08)	0.77 (0.60–0.98)	0.14
CD3^+^CD4^+^FOXP3^+b^ (regulatory)	1.00 (0.83–1.21)	0.83 (0.36–1.88)	0.97	1.13 (0.96–1.32)	0.86 (0.58–1.28)	0.39
CD3^+^TIM3^+b,d^ (exhausted)	1.45 (1.01–2.08)	0.43 (0.15–1.23)	0.11	0.92 (0.72–1.17)	0.75 (0.48–1.16)	0.61
CD3^+^PD1^+^TIM3^+b,d^ (terminally exhausted)	1.76 (1.11–2.78)	0.60 (0.15–2.37)	0.34	0.96 (0.70–1.32)	0.76 (0.42–1.36)	0.55
	**Order of first IUD use and first birth**^**c,f,h**^			**Order of first OC use and first birth**^**f,h**^		
	**Before first birth**	**After first birth**	***p***-**value**^**e**^	**Before first birth**	**After first birth**	***p***-**value**^**b**^
CD3^+^ (total)	1.03 (0.71–1.49)	1.09 (0.89–1.33)	0.78	1.09 (0.92–1.30)	1.05 (0.86–1.28)	0.76
CD3^+^CD4^+b,c^ (helper)	1.24 (0.89–1.73)	1.03 (0.86–1.22)	0.33	1.02 (0.87–1.20)	1.08 (0.90–1.28)	0.67
CD3^+^CD8^+b^ (cytotoxic)	1.13 (0.81–1.56)	1.18 (0.99–1.40)	0.81	1.08 (0.93–1.26)	1.04 (0.88–1.24)	0.76
CD3^+^CD4^+^FOXP3^+b^ (regulatory)	1.20 (0.81–1.79)	1.03 (0.84–1.27)	0.50	1.08 (0.89–1.30)	1.03 (0.83–1.27)	0.74
CD3^+^TIM3^+b,d^ (exhausted)	0.83 (0.24–2.86)	2.06 (1.31–3.22)	0.18	1.24 (0.87–1.76)	0.75 (0.46–1.24)	0.11
CD3^+^PD1^+^TIM3^+b,d^ (terminally exhausted)	0.44 (0.07–2.77)	2.01 (1.12–3.58)	0.12	1.35 (0.85–2.14)	0.68 (0.35–1.32)	0.10

^a^ORs were calculated using beta-binomial models using the positive number of cells for each cell type out of the total number of cells adjusting for age at cancer diagnosis, family history of breast or ovarian cancer in first degree relative (yes, no or not reported), study site (AACES, DOVE, HOPE, NECC, NHS, NHSII), histotype (high grade serous, low grade serous, mucinous, endometrioid, clear cell, other), and mutually adjusted for use of contraception method (never, ever).

^b^Additionally adjusted for tertile of total T cells (CD3^+^).

^c^Excludes AACES.

^d^Excludes DOVE and HOPE.

^e^Global Wald test.

^f^Excludes NHS.

^g^Dichotomized at median value.

^h^Parous women only.

^i^*p*-value for interaction term of parity with ever IUD or OC use.

-Model did not run due to small cell counts.

## Discussion

In this assessment of tumor immune profiles from 1800 ovarian cancer cases, we observed that ovarian tumor immune profiles may differ by method of contraception used earlier in life. Overall, infiltration of cytotoxic T cells (CD3^+^CD8^+^) in the tumor compartment was higher with prior IUD use and lower with previous OC use. There were also significantly higher odds of T cell exhaustion (CD3^+^TIM3^+^, CD3^+^PD1^+^TIM3^+^) with IUD use. These results suggest that prior IUD use may lead to a stronger overall tumor immune response among those who develop ovarian cancer. Results from stratified analyses suggested increased T cell exhaustion in relation to IUD use in the ovarian tumor microenvironment when individuals also had other inflammatory exposures, such as genital powder use and higher BMI.

The presence of a non-hormonal IUD in the uterus elicits a foreign body reaction that starts with inflammation followed by an influx of neutrophils, mononuclear cells, and plasma cells into the uterus and fallopian tubes, compromising the viability of sperm, oocytes, and embryos [8, 34–37]. Our findings suggest that immune responses due to IUD use could alter the tissue in such a way that may alter how the tumor itself develops and the associated anti-tumor immune response. We observed associations between IUD use and ovarian tumor T cells even though most women had not used an IUD for over 25 years. This suggests that IUDs can have long-lasting impacts on the female reproductive system.

IUDs, especially non-hormonal IUDs that were the dominant type of IUDs used in this study, can induce a local inflammatory response that results in activation of helper T cells (CD3^+^CD4^+^) [38]. A study comparing immune infiltrates in the fallopian tubes of women with and without IUDs revealed that IUD users had higher total leukocytes, granulocytes, and helper T cells (CD3^+^CD4^+^), suggesting local immunosurveillance is elevated with IUD use [36]. However, we did not observe any association of helper T cell tumor infiltration with IUD use in our study, and additional studies are needed to better characterize the effects of IUD use on helper T cells.

Studies of inflammation and IUD use have that found elevated inflammatory markers in local (uterine lavage, vaginal swab) samples from women after IUD insertion, including increased inflammatory cytokines in blood measured at 1- and 3-months following implantation that was greater among users of copper compared to hormonal IUDs [39, 40], but little or no differences in local and systemic (blood) samples long term [13, 15, 16]. In an assessment of women who had a solid organ transplant and healthy controls comparing uterine lavage and endometrial tissue samples taken before and after IUD insertion, investigators found increased TAM M1 phenotype activity following insertion, inciting a proinflammatory response and cytotoxic immune cell activation [41]. This inflammatory and immune response may accumulate with multiple IUD insertions, and this repeated exposure may lead to immune suppression and contribute to an exhaustion-like state of T cells in the uterine microenvironment [42]. In our study sample, each study site questionnaire varied and did not account for how many IUDs were used or how long each IUD was used; this variability may have contributed to the lack of differences observed in odds of T cell exhaustion by duration of IUD use. Although non-significant, we did observe greater T cell exhaustion among parous women who first used an IUD after their initial birth compared to before. This suggests that T cell exhaustion may be more prominent in women with ovarian cancer who had more recent exposure to IUD use and the subsequent inflammatory response. The inflammatory response associated with IUD use likely promotes progression towards T cell exhaustion that may have a synergistic effect among those who have precursor legions that develop into endometrioid and clear cell ovarian tumors [43]. While we did not have sufficient sample size to assess clear-cell specific associations, we did observe much higher odds of exhausted and terminally exhausted T cell infiltration (CD3+TIM3+, CD3+PD1+TIM3+) among endometrioid tumors. Furthermore, we also observed increasing odds of T cell exhaustion infiltrates associated with IUD use among those who had other pro-inflammatory exposures, including endometriosis, menopause, a BMI over 25 kg/m^2^, and smoking.

While OC use is hypothesized to have various effects on the immune system, the mechanisms through which this may occur are not established. Altered levels of circulating immune cells have been observed during periods of active OC use, such as higher cytotoxic T cells and lower regulatory T cells [44, 45]. There is less evidence supporting long-term immune changes associated with OC use at either the systemic or local level. However, investigations of inflammatory biomarkers associated with contraception use have shown there are implications of contraception use earlier in life that can be observed well past menopause [13, 46]. Several studies have observed increased levels of circulatory inflammatory markers [13, 47–50], which have the potential to impact the distribution of immune cells. The decreased cytotoxic T cells (CD3^+^CD8^+^) observed with oral contraception use suggest the presence of immunosuppressive inflammation. This may influence certain myeloid immune cells to downregulate anti-tumor immune responses, including cytotoxic T cells, and upregulate immunosuppressive cells that could facilitate tumor immune evasion [51–54]. Given that longer duration of OC use is associated with greater inflammation [55–57], it is not surprising that we observed lower odds of cytotoxic T cells with longer duration of OC use. Conversely, longer OC use results in fewer ovulatory cycles and therefore less local inflammation. However, when we restricted OC use to those who had used this method for at least five years, we did not observe any meaningful difference compared to the 1- year cut point presented in the results (data not shown). It is also important to note that our analysis was limited to ovarian cancer cases only, and we currently have no comparison of cytotoxic T cells in healthy ovarian tissue. While the specific mechanisms through which OCs may impact the immune environment are unclear, OC use has long-lasting effects on the risk of ovarian cancer [58, 59] and, in our study, on the anti-tumor immune response.

Potential limitations of our study include small numbers preventing histotype-specific stratified analyses, lack of diversity in the study population, age of tissue samples, lack of hormonal IUDs and residual confounding. Where possible, we presented histotype-specific results (Supplementary Table 4). The markers of T cell exhaustion (CD3^+^TIM3^+^, CD3^+^PD1^+^TIM3^+^) we used do not allow inference as to which T cell subsets (e.g. cytotoxic, helper) have been exhausted. Our sample includes tissue samples collected over decades, and it is possible that older tumor samples may have reduced antigenicity; however, an analysis by Hathaway and colleagues observed similar results when evaluating the overall study population and when limiting to samples <20 years old [7]. Furthermore, study sites within our sample began recruiting participants as early as 1976, so the type of IUD used is not likely to be hormonal, and therefore, results cannot be generalized to modern IUDs. Compared to hormonal IUDs, non-hormonal IUDs may be associated with a greater inflammatory response, leading to immune activation, although additional studies are needed to better characterize the differences in the localized response. Despite adjustment for other reproductive risk factors associated with contraception use, such as parity and other methods of contraception, residual confounding may remain, as we do not know the underlying reasons why some women may choose an IUD over OC. There may also be concerns of multiple testing; however, our analyses were planned to limit the number of tests to those relevant to our main objective regarding the associations of T cells with contraception use.

Our analysis had many strengths. While there is a substantial body of evidence demonstrating the effects of OCs on ovarian cancer, the long-term impact of IUD use has not been well-established, and no studies have looked at the impact of contraceptives such as IUD and OC use on tumor immunity. This analysis included many ovarian cancer cases from several studies across the US, increasing the reliability and generalizability of findings. The granularity in our approach to assessing IUD use by duration and timing allows for a more nuanced understanding of how IUD use may influence the tumor immune microenvironment. We also assessed multiple markers to meaningfully characterize tumor immune profiles. Although we were not able to report histotype-specific associations, our case-only analysis design allowed for the consideration of histotype in our models, resulting in a more accurate representation of the effects of contraception use on ovarian tumor T cells.

In summary, our findings suggest that past contraception use, often many years prior to diagnosis, has immunomodulatory effects on T cells in the ovarian tumor immune microenvironment among patients with ovarian cancer. Future research should evaluate how IUDs are associated with different tumor chrematistics (e.g., gene expression, methylation) that can impact the anti-tumor immune response. Additional research that includes more diverse populations and a greater array of immune markers is needed to better understand the impact of IUD use, including hormonal IUD use, on ovarian carcinogenesis. This is particularly important as contraception patterns are changing, and the consequences of the shift to increasing IUD use and decreasing OC use on ovarian cancer risk remain largely unknown.

## Supplementary information


Supplemental Table 1.
Supplemental Table 2.
Supplemental Table 3.
Supplemental Table 4.


## Data Availability

NHS/NHSII data cannot be publicly shared because of participant confidentiality and privacy concerns. According to standard controlled access procedures, applications to use NHS/NHSII resources will be reviewed by our External Collaborations Committee to verify that the proposed use maintains the protection of the privacy of participants and confidentiality of the data. Further information including the procedures to obtain and access data from the Nurses’ Health Studies is available at https://www.nurseshealthstudy.org/researchers (contact email: nhsaccess@channing.harvard.edu). AACES and NECC data that support the findings of this study are available upon request and reviewed by the study leadership. Applications to use DOVE data can be submitted according to standard controlled access procedures by contacting the DOVE PIs. Other data generated in this study are available upon reasonable request from the corresponding author. The lead author (JMM) confirms that the manuscript is an honest, accurate, and transparent account of the study being reported and that no important aspects of the study have been omitted.

## Code availability

The code used to generate the results in this study is available from the corresponding author upon reasonable request.
