# Supplementary material for: Ovarian cancer tumor immune profiles associated with intrauterine device and oral contraceptive use
Source: Br J Cancer. 2026 Jun 27;135(6):972–82. doi: 10.1038/s41416-026-03508-9 (PMC13534713; doi:10.1038/s41416-026-03508-9)
Supplement: Supplementary file 1 [file 41416_2026_3508_MOESM1_ESM.docx]

**Supplemental Table 1**. Description of antibodies and samples used to measure tumor T cells among AACES, DOVE, HOPE, NECC, NHS, and NHSII participants (n=1,800)^a^

| **T Cell** | **Antibody** | **Panel** | **Vendor** | **Clone** | **Isotype** | **Species** | **Catalogue  number** | **Site(s)** | **Cases (n)** |
| --- | --- | --- | --- | --- | --- | --- | --- | --- | --- |
| Total | CD3 | 31 | ThermoFisher | SP7 | IgG | Rb Monoclonal | MA5-14524 | AACES, DOVE, HOPE, NECC, NHS, NHSII | 1,751 |
| Helper | CD4 | 31 | Cell Marque | EP204 | IgG | Rb Monoclonal | 104R-25 | DOVE, HOPE, NECC, NHS, NHSII | 1,654 |
| Cytotoxic | CD8alpha | 31 | DAKO | C8/144B | IgG1 | Ms Monoclonal | M7103 | AACES, DOVE, HOPE, NECC, NHS, NHSII | 1,751 |
| Regulatory | FOXP3 | 31 | Abcam | 236/E7 | IgG1 | Ms Monoclonal | ab20034 | AACES, DOVE, HOPE, NECC, NHS, NHSII | 1,751 |
| Exhausted | TIM3 | 12 | CST | (D5D5R) | IgG | Rb Monoclonal | 45208 | AACES, NECC, NHS, NHSII | 672 |
| Terminally exhausted | PD1 | 12 | CST | D4W2J | IgG | Rb Monoclonal | 86163 S | AACES, NECC, NHS, NHSII | 672 |
