# Supplementary material for: Ovarian cancer tumor immune profiles associated with intrauterine device and oral contraceptive use
Source: Br J Cancer. 2026 Jun 27;135(6):972–82. doi: 10.1038/s41416-026-03508-9 (PMC13534713; doi:10.1038/s41416-026-03508-9)
Supplement: Supplementary file 2 [file 41416_2026_3508_MOESM2_ESM.docx]

**Supplemental Table 2**. Odds ratios (OR) and 95% confidence intervals (CI) for positivity of tumor immune infiltrations associated with ever use of intrauterine device (IUD) or oral contraception (OC) among participants by study site (n=1,800)^a^

|  | **CD3^+^ (total)** | | **CD3^+^CD4^+c,d^  (helper)** | | **CD3^+^CD8^+c^  (cytotoxic)** | |
| --- | --- | --- | --- | --- | --- | --- |
|  | **Ever IUD  vs. never** | **Ever OC ≥1 year  vs. never** | **Ever IUD  vs. never** | **Ever OC ≥1 year  vs. never** | **Ever IUD  vs. never** | **Ever OC ≥1 year  vs. never** |
| AACES (n=125) | 0.95 (0.38-2.37) | 1.02 (0.61-1.71) | na^d^ | na^d^ | 1.82 (0.71-4.69) | 0.76 (0.44-1.32) |
| DOVE (n=862) | 1.07 (0.86-1.32) | 0.97 (0.80-1.18) | 0.98 (0.84-1.15) | 1.01 (0.87-1.16) | 1.11 (0.92-1.32) | 0.98 (0.82-1.17) |
| HOPE (n=45) | 0.67 (0.24-1.85) | 0.32 (0.14-0.74) | - | - | - | - |
| NECC (n=389) | 0.86 (0.62-1.20) | 1.05 (0.81-1.37) | 1.32 (0.88-1.96) | 0.99 (0.72-1.35) | 1.16 (0.88-1.52) | 0.96 (0.78-1.19) |
| NHS (n=292) | 1.30 (0.63-2.70) | 0.90 (0.63-1.29) | 1.46 (0.61-3.47) | 1.53 (1.00-2.33) | 1.44 (0.80-2.60) | 0.76 (0.56-1.03) |
| NHSII (n=87) | 3.23 (0.26-39.73) | 0.93 (0.46-1.90) | 4.99 (0.71-34.86) | 1.44 (0.75-2.77) | 0.85 (0.17-4.34) | 0.68 (0.41-1.15) |
| *p*-het^b^ | 0.68 | 0.20 | 0.17 | 0.23 | 0.79 | 0.42 |
|  | **CD3^+^CD4^+^FOXP3^+c^  (regulatory)** | | **CD3^+^TIM3^+c,e^  (exhausted)** | | **CD3^+^PD1^+^TIM3^+c,e^  (terminally exhausted)** | |
|  | **Ever IUD  vs. never** | **Ever OC ≥1 year  vs. never** | **Ever IUD  vs. never** | **Ever OC ≥1 year  vs. never** | **Ever IUD  vs. never** | **Ever OC ≥1 year  vs. never** |
| AACES (n=125) | 0.24 (0.08-0.69) | 1.22 (0.78-1.89) | 0.90 (0.42-1.92) | 0.72 (0.44-1.17) | 1.62 (0.69-3.80) | 0.96 (0.54-1.70) |
| DOVE (n=862) | 1.03 (0.84-1.27) | 0.99 (0.82-1.20) | na^e^ | na^e^ | na^e^ | na^e^ |
| HOPE (n=45) | - | - | na^e^ | na^e^ | na^e^ | na^e^ |
| NECC (n=389) | 1.20 (0.78-1.86) | 1.08 (0.76-1.52) | 1.48 (1.00-2.18) | 0.88 (0.64-1.21) | 1.49 (0.88-2.53) | 0.92 (0.60-1.41) |
| NHS (n=292) | 1.19 (0.38-3.70) | 1.55 (0.91-2.64) | 1.31 (0.56-3.04) | 0.91 (0.60-1.38) | 1.82 (0.62-5.39) | 0.93 (0.53-1.64) |
| NHSII (n=87) | 0.85 (0.17-4.34) | 0.68 (0.41-1.15) | - | - |  |  |
| *p*-het^b^ | 0.92 | 0.19 | 0.54 | 0.71 | 0.94 | 0.99 |

Abbreviations: CI, confidence interval; AACES, African-American Cancer Epidemiology Study; DOVE, Diseases of the Ovary and their Evaluation; HOPE, Hormones and Ovarian Cancer Prediction Study; NECC, New England Case Control Study; NHS, Nurses’ Health Study

^a^ ORs were calculated using beta-binomial models using the positive number of cells for each cell type out of the total number of cells adjusting for age at cancer diagnosis, parity (never pregnant, 1 full term birth, 2 or more full term births), family history of breast or ovarian cancer in first degree relative (yes, no or not reported), histotype (high grade serous, low grade serous, mucinous, endometrioid, clear cell, other), study site (AACES, DOVE, HOPE, NECC, NHS, NHSII), and mutually adjusted for use of contraception method (never, ever)

^b^ Test for heterogeneity using Cochrane’s Q *p*-value

^c^ Additionally adjusted for tertile of total T cells (CD3^+^)
^d^ Excludes AACES
^e^ Excludes DOVE and HOPE

- Model did not run due to small cell counts
