# Supplementary material for: Ovarian cancer tumor immune profiles associated with intrauterine device and oral contraceptive use
Source: Br J Cancer. 2026 Jun 27;135(6):972–82. doi: 10.1038/s41416-026-03508-9 (PMC13534713; doi:10.1038/s41416-026-03508-9)
Supplement: Supplementary file 3 [file 41416_2026_3508_MOESM3_ESM.docx]

**Supplemental Table 3**. Odds ratios (OR) and 95% confidence intervals (CI) for positivity of tumor immune infiltrations associated with exclusive use of intrauterine device (IUD) or oral contraception (OC) compared to never among AACES, DOVE, HOPE, NECC, NHS, and NHSII participants (n=1,800)^a^

|  | **Ever used IUD only n=121** | **Ever used OC ≥1 year only**  **n=731** | **Ever used IUD and OC ≥1 year**  **n=192** |
| --- | --- | --- | --- |
| **T cell infiltration** |  |  |  |
| CD3^+^ (total) | 0.92 (0.72-1.19) | 0.95 (0.82-1.09) | 1.00 (0.80-1.24) |
| CD3^+^CD4^+b,c^ (helper) | 1.14 (0.89-1.46) | 1.09 (0.94-1.26) | 1.11 (0.90-1.37) |
| CD3^+^CD8^+b^ (cytotoxic) | 0.97 (0.78-1.22) | 0.86 (0.75-0.97) | 1.09 (0.90-1.32) |
| CD3^+^CD4^+^FOXP3^+b^ (regulatory) | 0.99 (0.74-1.33) | 1.07 (0.91-1.26) | 1.03 (0.81-1.32) |
| CD3^+^TIM3^+b,d^ (exhausted) | 1.86 (1.15-3.00) | 0.97 (0.77-1.22) | 0.91 (0.57-1.44) |
| CD3^+^PD1^+^TIM3^+b,d^ (terminally exhausted) | 2.19 (1.20-4.02) | 1.01 (0.75-1.36) | 1.06 (0.59-1.94) |

Abbreviations: CI, confidence interval; AACES, African-American Cancer Epidemiology Study; DOVE, Diseases of the Ovary and their Evaluation; HOPE, Hormones and Ovarian Cancer Prediction Study; NECC, New England Case Control Study; NHS, Nurses’ Health Study

^a^ ORs were calculated using beta-binomial models using the positive number of cells for each cell type out of the total number of cells adjusting for age at cancer diagnosis, parity (never pregnant, 1 full term birth, 2 or more full term births), family history of breast or ovarian cancer in first degree relative (yes, no or not reported) histotype (high grade serous, low grade serous, mucinous, endometrioid, clear cell, other), study site (AACES, DOVE, HOPE, NECC, NHS, NHSII), and mutually adjusted for use of contraception method (never, ever)

^b^ Additionally adjusted for tertile of total T cells (CD3^+^)
^c^ Excludes AACES
^d^ Excludes DOVE and HOPE
